# Multiscale Topology Design Based on Non-Penalisation Smooth-Edged Material Distribution for Optimising Topology (SEMDOT)

**DOI:** 10.3390/ma18102394

**Published:** 2025-05-20

**Authors:** Jiye Zhou, Yun-Fei Fu, Kazem Ghabraie

**Affiliations:** 1 School of Engineering, Deakin University, Geelong, VIC 3216, Australia; zhoujiy@deakin.edu.au; 2 Department of Mechanical Engineering, University of Alberta, Edmonton, AB T6G 1H9, Canada; yfu15@ualberta.ca

**Keywords:** SEMDOT, non-penalisation method, multiscale design, smooth boundary

## Abstract

This study presents an extension of the Smooth-Edged Material Distribution Optimisation Technique (SEMDOT) to multiscale topology optimisation (MSTO). While the SEMDOT has shown promise in producing smooth and fabrication-friendly structures in various single-scale problems, its application to multiscale design remains unexplored. To extend SEMDOT to MSTO, a discrete sensitivity approach without penalisation is introduced, in which sensitivities are directly determined by classifying elements. Microstructural properties are computed using energy-based homogenisation with periodic boundary conditions (PBCs), enabling efficient and accurate prediction of effective elastic moduli. Physical fidelity of the smooth boundaries estimated by level-set functions are validated. Numerical results from 2D and 3D compliance minimization benchmarks demonstrate the effectiveness of the SEMDOT method, resulting in smooth boundaries between solid and void phases at both macro- and microscales, overcoming the jagged boundaries and grayscale issues seen in conventional methods. The results also show that the SEMDOT achieves comparable performance to other MSTO methods, with fewer iterations and reduced computational time.

## 1. Introduction

Topology optimisation (TO) is a powerful structural design method that seeks the optimal material distribution within a given domain under specified loads and constraints [1]. Compared to traditional shape and size optimisation, TO offers greater flexibility and enables the creation of highly efficient structures, making it widely adopted in automotive, aerospace, and civil engineering applications [2]. Over the past decades, several TO methods have been developed. Conventional density-based methods, such as solid isotropic material penalisation (SIMP) [3,4] and bi-directional evolutionary structural optimisation (BESO) [5,6], have been widely adopted. However, density-based methods often suffer from numerical instabilities that result in blurred or jagged structural boundaries. Normally, additional post-processing is required for the topological structures.

To form smooth and clear boundaries, the level-set method (LSM) employs the zero-level set of high-dimensional functions to define structural boundaries [7]. Smooth and well-defined boundaries are obtained by solving the Hamilton–Jacobi equation. Moving morphable component (MMC) and moving morphable void (MMV) methods are proposed in [8,9], where smooth boundaries can be explicitly represented by geometric components or voids with fewer design variables. Furthermore, the phase-field method [10] models the design domain using phase-field functions and evolves the interface by solving the Allen–Cahn equation, which naturally captures the diffusive characteristics of material boundaries and produces smooth boundary transitions. The H^1^ gradient method [11] achieves smooth deformation of both boundary and interior nodes by applying shape gradients as distributed loads on the design boundary and solving an auxiliary linear elasticity problem, thus avoiding jagged geometries without requiring mesh remeshing. Despite these methods’ ability to produce smooth boundaries, these methods come with limitations. The performance of the LSM and MMCs is often sensitive to the initial design configuration, leading to non-robust outcomes across different starting conditions. The phase-field method is highly sensitive to parameters such as interface thickness and control coefficients. The H^1^ gradient method incurs a higher computational cost compared to conventional approaches, as it requires integration of both functions and their first-order derivatives.

To overcome these limitations, the Smooth-Edged Material Distribution Optimisation Technique (SEMDOT) was recently introduced as a newly developed density-based method incorporating level-set functions for the design of smooth and fabrication-friendly structures [12]. In this approach, elemental volume fractions are chosen as design variables instead of element densities. These volume fractions are eventually represented as explicit and smooth boundaries by comparing densities of interpolated grid points within both macro- and microelements to predefined threshold level-set values. As a density-based method, the SEMDOT maintains the simplicity of sensitivity analysis without the need for penalisation or extra control parameters, while also offering greater design flexibility and reduced dependence on the initial layout.

The effectiveness of the SEMDOT has been validated across a range of applications. It has been successfully applied to the design of self-supporting structures for additive manufacturing [13], achieving well-performed structures in solving the natural frequency maximisation problem [14]. It has also demonstrated accurate boundary resolution in heat convection problems [15] and has been tested against real-world nonlinear structural benchmarks involving structural steel [16]. However, the SEMDOT has so far only been applied to shear modulus maximisation in microstructures, and its application in multiscale topology optimisation (MSTO) remains largely unexplored.

Unlike single-scale design, which focuses solely on either the macro- or micro-level, MSTO draws inspiration from biological materials such as bones and plant leaves, which achieve remarkable strength and adaptability through intricate microstructures [17]. This approach explicitly couples microscale design with macroscopic optimisation, enabling the creation of functionally graded materials with improved structural performance [18]. To analyse the interaction between micro- and macroscales, homogenisation methods are commonly employed [19].

The traditional numerical homogenisation method estimates effective material properties by solving boundary value problems on a representative volume element (RVE) at each design point [20]. To reduce the associated computational cost, multiscale design frameworks often adopt predefined or parameterised microstructural unit cells. For instance, Wu et al. [21] proposed a surrogate model that maps the effective properties of density-parameterised unit cells. Common parameterised geometries, such as X-shaped and plus-shaped cells with independently controlled thicknesses [22], or 3D cubic RVEs defined by seven independent parameters [23]. To further enhance structural diversity, Feng et al. [24] developed large datasets of boundary-identical microstructures using active learning strategies, while Zhai et al. [25] introduced a differentiable transformation approach, in which an initial low-density microstructure evolves through non-uniform geometric changes to produce a continuum of higher-density designs. More recently, the Multiple Variable Cutting (M-VCUT) LSM has been proposed to generate spatially varying cellular microstructures by superimposing multiple cutting functions on level set fields [26,27]. Despite these advances, many of these methods rely heavily on predefined base shapes or tightly controlled parameters, which limits the design flexibility.

Compared to traditional numerical homogenisation [28], the energy-based homogenisation method employing the average stress and strain theorem offers better computational efficiency. It has also been identified as an equivalent approach for the prediction of effective material properties compared to the asymptotic approach [29]. Xia and Breitkopf [30] demonstrated the effectiveness of this approach in optimising porous materials with tailored elastic properties, enabling designs that achieve near-optimal compliance. Zhang and Khandelwal [31] applied energy-based homogenisation to generate microstructures with programmable anisotropy in lightweight designs. To ensure the accuracy of estimating properties from micro- to macroscales, periodic boundary conditions (PBCs) should be applied, where the displacement field on opposite boundaries of the representative volume element (RVE) should be constrained to be equal but offset by a constant that represents macroscopic strain [32].

More recently, the multiscale design has been integrated into different TO methods. Gao et al. [33] proposed the multiscale design based on the SIMP method, while Kazakis and Lagaros [34] integrated the multiscale design into the BESO method. However, grayscale elements or zig-zag boundaries might emerge in the results. To alleviate these issues, an additional thresholding process might be required [35]. Apart from the density-based method, the isogeometrical (IGA) level-set approach has also been integrated into the multiscale design [36], and smooth boundaries in the final layouts of both scales are obtained. However, the simultaneous optimisation of macro- and microstructures using level-set methods and an IGA approach can be computationally intensive.

This study aims to extend the SEMDOT method to MSTO based on energy homogenisation method, leveraging its advantages in smooth boundary representation and straightforward sensitivity analysis. The key innovation lies in replacing conventional continuous sensitivity formulations with a discrete sensitivity scheme that avoids penalisation, wherein sensitivities are directly assigned based on the classification of elements as solid, void, or boundary [37,38,39,40]. To validate the physical accuracy of the smooth designs generated by level-set functions, a remeshing strategy [41] is implemented to obtain more precise homogenised elastic properties. The proposed approach is demonstrated to achieve results comparable to those of existing state-of-the-art MSTO methods. Its effectiveness is further validated through a series of 2D and 3D compliance-based benchmark problems.

The remainder of this paper is structured as follows: Section 2 details the framework of the non-penalisation SEMDOT method. Section 3 explains the integration of homogenisation methods, especially for the sensitivity analysis and convergence criteria. Section 4 demonstrates numerical results based on different 2D and 3D compliance-based design problems. Finally, the conclusion is drawn in Section 5.

## 2. Framework of Non-Penalisation SEMDOT Method

In the SIMP method, intermediate elements with densities between 0 and 1 are driven toward a binary design through the application of penalty factors. In contrast, the non-penalisation SEMDOT approach eliminates the penalisation process, where the calculation of Young’s modulus can be directly formulated as

(1) $$E_{e}\left(\rho_{e,n}\right)=\rho_{e,n}E_{s}$$

where $E_{e}\left(\rho_{e,n}\right)$ is Young’s modulus as a function of grid point densities, $E_{s}$ is Young’s modulus of solid materials, and $\rho_{e,n}$ is the density of the nth grid point in the eth element.

**Figure 1:**
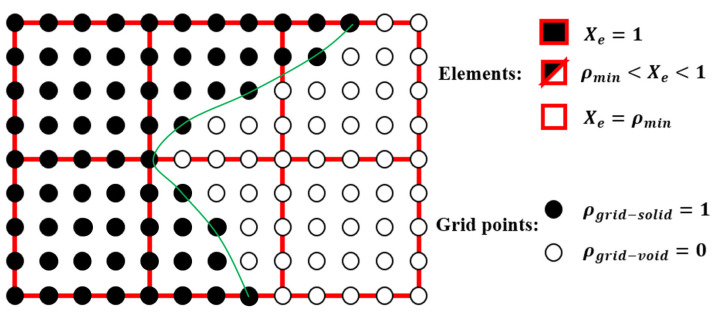
An illustration of the SEMDOT method.

During the optimisation process, intermediate elements will be linearly interpolated to grid points. The densities of grid points are represented as void or solid with discrete values of 0 or 1, as shown in Figure 1. To comply with the finite element calculation that is implemented on an element level, an element volume fraction variable depending on the grid points’ densities will be expressed as a surrogate function such that

(2) $$\Psi_{e}=\frac{1}{N}\sum_{n=1}^{N}\rho_{e,n}$$

where $\Psi_{e}$ is the elemental volume fraction, and N is the total number of grid points in each element. In this way, the discrete values of $\rho_{e,n}$ could be transformed into a new continuous elemental volume fraction variable $\Psi_{e}$ with values ranging from 0.001 (void elements) to 1 (solid elements).

Combining Equations (1) and (2), it is not hard to conclude that the stiffness matrix of a solid or void element in non-penalisation SEMDOT is the following:

(3) $$E_{e}\left(\Psi_{e}\right)=\Psi_{e}E_{s}$$

Similarly, the stiffness matrix of the element e could be given as follows:

(4) $$K_{e}\left(\Psi_{e}\right)=\Psi_{e}K^{1}$$

where $K^{1}$ is the stiffness matrix of the solid element.

As mentioned before, the intermediate densities will be linearly interpolated, so the element stiffness matrix could also be explained as

(5) $$\begin{array}{c} K_{e}\left(\Psi_{e}\right)=\left(1-\Psi_{e}\right)K_{v}+\Psi_{e}K^{1}\\ =\left(1-\Psi_{e}\right)\rho_{min}K^{1}+\Psi_{e}K^{1} \end{array}$$

where $K_{v}$ is the stiffness matrix of the void element, which could also be expressed as the multiplication of the minimum density $\rho_{min}$ and stiffness matrix of the solid element.

During the optimisation process, two filters are utilised to control the feature size; one is the filter that is applied at the element level:

(6) $$\begin{array}{c} \tilde{\Psi_{e}}=\frac{\sum_{l=1}^{N_{e}}\omega_{el}\Psi_{l}}{\sum_{l}^{N_{e}}\omega_{el}}\;\\ \omega_{el}=\max\left(0,r_{min}-\Delta \left(e,l\right)\right) \end{array}$$

where $\tilde{\Psi_{e}}$ represents the filtered density of the eth element. $N_{e}$ denotes the set of neighbouring elements located within the filtering domain, which is defined as a circular region centred at the centroid of the element e with a specified filter radius $r_{min}$. The weighting factor $\omega_{el}$ is determined using a max-based operator, and $\Delta \left(e,l\right)$ is the distance between the centre of an element e and the lth element.

The other filter is heuristic [42], which is applied to transfer the element density into nodal density:

(7) $$\begin{array}{c} \rho_{n}=\frac{\sum_{e=1}^{M}\omega_{ne}\tilde{\Psi_{e}}}{\sum_{e=1}^{M}\omega_{ne}}\\ \omega_{ne}=\max\left(0,r_{nmin}-\Delta \left(n,e\right)\right) \end{array}$$

where $\rho_{n}$ is the nodal density of nth node, M is the total number of elements, $\omega_{ne}$ is the weighting factor, which is defined by a max operator, $r_{nmin}$ is the filter radius for the node, and $\Delta \left(n,e\right)$ is the distance between the centre of the element e and the nth node.

Then, the nodal densities will be linearly interpolated as grid point densities through the shape function $N^{\varepsilon }$:

(8) $$\begin{array}{c} \rho \left(\xi ,\eta \right)=\sum_{\varepsilon =1}^{4}N^{\varepsilon }\left(\xi ,\eta \right)\rho_{n}^{\varepsilon },\rho \left(\xi ,\eta \right)\in \rho \left(x,y\right)\\ N^{\varepsilon }\left(\xi ,\eta \right)=\frac{1}{4}\left(\left(1+\xi_{o}\xi_{\varepsilon }\right)+\left(1+\eta_{o}\eta_{\varepsilon }\right)\right) \end{array}$$

where $\left(\xi ,\eta \right)$ is the local coordinate of the grid point, $\rho_{n}^{\varepsilon }$ is the density for the εth node of the element, and $N^{\varepsilon }\left(\xi ,\eta \right)$ is an appropriate shape function. $\xi_{\varepsilon }$ and $\eta_{\varepsilon }$ are the local coordinates of the εth node, and $\xi_{o}$ and $\xi_{o}$ are the non-dimensional coordinates of the grid point o.$\;\rho \left(x,\;y\right)$ is the density of the grid point at global coordinate $\left(x,\;y\right)$.

Finally, a smooth Heaviside function will be used to obtain the solid/void grid points, as shown in Figure 1, which is

(9) $$\rho_{e,n}=\frac{\tanh\left(\beta \cdot \varphi \right)+\tan h\left[\beta \cdot \left(\rho \left(x,y\right)-\varphi \right)\right]}{\tanh\left(\beta \cdot \varphi \right)+\tan h\left[\beta \cdot \left(1.0-\varphi \right)\right]}$$

where Ψ is a threshold value defined by the bi-section method, and β is a scaling parameter that controls the steepness and is updated by the following:

(10) $$\beta_{k}=\beta_{k-1}+\delta$$

where *k* represents the current iteration number. The parameter β is initially set to 0.5 and is updated in each iteration with an evolution rate *δ* of 0.5.

The smooth topological boundary is implicitly represented via a level-set function $\Phi \left(x,\;y\right)$:

(11) $$\Phi \left(x,\;y\right)=\left\{\begin{array}{c} (\rho \left(x,y\right)-\varphi >0\;\mathrm{for}\;\mathrm{solid}\;\mathrm{region}\\ (\rho \left(x,y\right)-\varphi =0\;\;\;\mathrm{for}\;\mathrm{boundary}\\ (\rho \left(x,y\right)-\varphi <0\;\;\;\mathrm{for}\;\mathrm{void}\;\mathrm{region} \end{array}\right.$$

where $\Phi \left(x,\;y\right)$ is the level-set value for each grid point, and φ is the floating value that has been determined using a bi-section method.

Due to the fact that the design variables are not penalised, the resulting topologies often contain a large number of intermediate elements, which leads to a formulation resembling the classical “variable-thickness-sheet” problem [43], which is known to be a convex optimisation problem.

To address the challenges associated with intermediate densities, a discrete sensitivity approach proposed by Liang et al. [37,38,39,40] is adopted, which can be formulated as follows:

(12) $$\frac{\partial C\left(\Psi_{e}\right)}{\partial \Psi_{e}}=\left\{\begin{array}{c} \frac{C\left(\Psi_{e}=1\right)-C\left(\Psi_{e}=0\right)}{1}\approx -u_{e}^{T}K^{1}u_{e}\;\;\;\;\;\;if\;\Psi_{e}=1,\\ \;\frac{C\left(\Psi_{e}=0\right)-C\left(\Psi_{e}=1\right)}{-1}\approx -\rho_{min}u_{e}^{T}K^{1}u_{e}\;\;\;\;\;\;if\;\Psi_{e}=\rho_{min}.\; \end{array}\right.$$

where C is the objective (compliance) function, $\Psi_{e}$ is the elemental volume fraction of the eth element’s solid region, with $\Psi_{e}=1$ representing a solid element and $\Psi_{e}=\rho_{min}$ indicating a void element. $u_{e}$ represents the displacements of the eth element.

This method recalculates sensitivities without relying on penalisation, as the penalty factor p is entirely excluded from the formulation. The treatment of void elements in this formulation is heuristic, but the discrete sensitivities effectively differentiate between solid and void elements based on their influence on structural performance. The accuracy and robustness of this method have been both analytically and numerically demonstrated in [40,41].

Unlike continuous sensitivity formulations, which apply uniformly across all elements, the discrete variable sensitivity method is rooted in discrete optimisation algorithms such as sequential approximate integer programming. As such, it does not impose a fixed form for handling intermediate elements. Based on the discrete formulation in Equation (12), the sensitivities of intermediate elements in the non-penalisation SEMDOT can be consistently derived as follows:

(13) $$\begin{array}{c} \frac{\partial C\left(\Psi_{e}\right)}{\partial \Psi_{e}}=\left(1-\Psi_{e}\right){\left.\frac{\partial C\left(\Psi_{e}\right)}{\partial \Psi_{e}}\right|}_{\Psi_{e}=\rho_{min}}+\Psi_{e}{\left.\frac{\partial C\left(\Psi_{e}\right)}{\partial \Psi_{e}}\right|}_{\Psi_{e}=1}\\ =-\left[\left(1-\Psi_{e}\right)\rho_{min}+\Psi_{e}\right]u_{e}^{T}K^{1}u_{e} \end{array}$$

where $\left(1-\Psi_{e}\right)$ means the elemental volume fraction of the *e*th element’s void region, ${\left.\partial C\left(\Psi_{e}\right)/\partial \Psi_{e}\right|}_{\Psi_{e}=\rho_{min}}$ ($\rho_{min}$ is commonly defined as 1 × 10^−6^) represents the sensitivity of void elements, and ${\left.\partial C\left(\Psi_{e}\right)/\partial \Psi_{e}\right|}_{\Psi_{e}=1}$ represents the sensitivity of solid elements.

## 3. Integration of Homogenisation Method into the SEMDOT Method

The homogenisation approach is widely adopted in multiscale design and relies on two fundamental assumptions: (1) the characteristic size of the microstructures is significantly smaller than that of the macrostructure, and (2) the microstructures exhibit periodic distribution throughout the domain.

For the traditional homogenisation method, asymptotic expansion theory is used to describe the displacement field $u^{\epsilon }\left(x\right)$:

(14) $$u^{\epsilon }\left(x\right)=u_{0}\left(x,y\right)+\epsilon u_{1}\left(x,y\right)+\epsilon^{2}u_{2}\left(x,y\right)+\cdots$$

where ϵ represents the scaling factor (aspect ratio) between macro- and microstructures.

In the energy-based homogenisation approach, it is common to consider only the first-order variation in the displacement field. Based on this assumption, the macroscopic stiffness tensor $D^{H}$ can be derived by computing the strain energy density, i.e., the total strain energy divided by the area in 2D cases or the volume in 3D cases.

(15) $$D^{H}=\frac{1}{\left|\Omega_{m}\right|}\int_{\Omega_{m}}D\left(\varepsilon \left(u_{m}^{0}\right)-\varepsilon \left(u_{m}\right)\right)\left(\varepsilon \left(u_{m}^{0}\right)-\varepsilon \left(u_{m}\right)\right)d\Omega_{m}$$

where $\Omega_{m}$ denotes the area (in 2D) or volume (in 3D) of the microstructural domain, $\varepsilon \left(u_{m}^{0}\right)$ represents the prescribed unit-test strain field as defined in [44], and $\varepsilon \left(u_{m}\right)$ corresponds to the resulting strain field within the microstructure.

Due to the finite element discretisation used in the SEMDOT framework, the homogenised elastic tensor in Equation (15) can be reformulated as follows:

(16) $$D_{ijkl}^{H}=\frac{1}{\left|\Omega_{m}\right|}\sum_{e=1}^{N}{\left(u_{e}^{A\left(ij\right)}\right)}^{T}K^{1}u_{e}^{A\left(kl\right)}$$

where N denotes the total number of microscale elements contained within a single macroscale unit cell, $u_{e}^{A\left(ij\right)}\;$ and $\;u_{e}^{A\left(kl\right)}$ are the elemental displacement solutions corresponding to the three unit-test strain fields (in 2D): normal strain in the *x*-direction, normal strain in the *y*-direction, and shear strain.

To ensure that the deformation of adjacent unit cells is consistent and avoid artificial stress concentrations or discontinuities at the boundaries, PBCs are implemented when estimating the displacement fields of microstructures. The idea is to assume that the representative volume element (RVE) represents a repeating unit within an infinite periodic microstructure. Under PBCs, the displacement field on one side of the RVE must match the displacement on the opposite side, offset by a prescribed macroscopic strain, as shown in [30].

### 3.1. Sensitivity Analysis

Returning to the compliance-based macroscopic structural problem, the design variables at both the micro- and macroscales can be formulated as follows:

(17) $$C\left(\Psi_{M},\Psi_{m}\right)=\sum_{i=1}^{N}U_{i}^{T}K_{i}\left(\Psi_{M},\Psi_{m}\right)U_{i}$$

where $\Psi_{M},\Psi_{m}$ are the macroscopic and microscopic elemental volume fractions, respectively. $U_{i}$ and $K_{i}$ represent the nodal displacement and the stiffness matrix of the ith element with respect to the macrostructure, with the total number of elements equal to N.

The general form of the elemental stiffness matrix is taken in the following form:

(18) $$K=\int_{V}B^{T}DBdV$$

where B is the strain displacement matrix, and D is the elastic tensor of the element.

For microstructures, the elastic tensor $D_{m}$ is formulated to comply with the non-penalisation SEMDOT method based on the base material $D_{0}$:

(19) $$D_{m}=[\Psi_{m}+\left(1-\Psi_{m}\right)\rho_{min})]D_{0}$$

Then, the macroscopic elastic tensor $D_{M}$ can be obtained from $D^{H}$ and also be expressed in a non-penalisation SEMDOT mode:

(20) $$D_{M}=[\Psi_{M}+\left(1-\Psi_{M}\right)\rho_{min})]D^{H}$$

For compliance-based problems, the sensitivity for the objective and constraint concerning the design variable of the macroscopic cell can be illustrated as

(21) $$\begin{array}{c} \frac{\partial C\left(\Psi_{M}\right)}{\partial \Psi_{M}}=\left(1-\Psi_{M}\right){\left.\frac{\partial C\left(\Psi_{M}\right)}{\partial \Psi_{M}}\right|}_{\Psi_{M}=\rho_{min}}+\Psi_{M}{\left.\frac{\partial C\left(\Psi_{M}\right)}{\partial \Psi_{M}}\right|}_{\Psi_{M}=1}\\ =-\left[\left(1-\Psi_{M}\right)\rho_{min}+\Psi_{M}\right]U_{M}^{T}\int_{\Omega_{M}}B^{T}D^{H}\left(\Psi_{m}\right)Bd\Omega_{M}U_{M}\\ \frac{\partial V_{M}\left(\Psi_{M}\right)}{\partial \Psi_{M}}=\frac{\partial \Psi_{M}V_{0}}{\partial \Psi_{M}}=1 \end{array}$$

where $V_{M}\left(\Psi_{M}\right)$ is the elemental volume fraction of the Mth macroelement, which can be calculated by the multiplication of $\Psi_{M}$ with a unit volume of the element ($V_{0}=1)$.

The derivation of the first-order derivatives of the objective function and the microvolume constraint for the microdesign variables can be given as

(22) $$\begin{array}{c} \frac{\partial C\left(\Psi_{m}\right)}{\partial \Psi_{m}}=-\left[\left(1-\Psi_{m}\right)\rho_{min}+\Psi_{m}\right]U_{M}^{T}\int_{\Omega_{m}}B^{T}\frac{\partial D^{H}\left(\Psi_{m}\right)}{\partial \Psi_{m}}Bd\Omega_{m}U_{M}\\ \frac{\partial V_{e}\left(\Psi_{m}\right)}{\partial \Psi_{m}}=\frac{\partial \Psi_{m}V_{0}}{\partial \Psi_{m}}=1 \end{array}$$

where $V_{m}\left(\Psi_{m}\right)$ is the elemental volume fraction of the mth microelements.

The first-order derivatives of the homogenised elastic tensor $D^{H}$ with respect to the microelement densities are given as follows:

(23) $$\begin{array}{c} \frac{\partial D^{H}\left(\Psi_{m}\right)}{\partial \Psi_{m}}=\left(1-\Psi_{m}\right){\left.\frac{\partial D^{H}\left(\Psi_{m}\right)}{\partial \Psi_{m}}\right|}_{\Psi_{m}=\rho_{min}}+\Psi_{m}{\left.\frac{\partial D^{H}\left(\Psi_{m}\right)}{\partial \Psi_{m}}\right|}_{\Psi_{m}=1}\\ =-\frac{1}{2}\int_{\Omega_{m}}\left[\left(1-\Psi_{m}\right)\rho_{min}+\Psi_{m}\right]D_{ijkl}^{0}\left(\varepsilon \left(u_{m}^{0}\right)-\varepsilon \left(u_{m}\right)\right)\left(\varepsilon \left(u_{m}^{0}\right)-\varepsilon \left(u_{m}\right)\right)d\Omega_{m} \end{array}$$

The developed method can be smoothly and efficiently solved using gradient-based MMA algorithms [45].

### 3.2. Convergence Criteria

The convergence criteria of multiscale design in the SEMDOT are defined by two constraints. One is the overall topological alteration, which is defined as

(24) $$\left\{\begin{array}{c} \frac{\sum_{M=1}^{N_{M}}\left|\Psi_{M}^{k}-\Psi_{M}^{k-1}\right|}{\sum_{M=1}^{N_{M}}\Psi_{M}}”\leq \tau \\ \frac{\sum_{m=1}^{N_{m}}\left|\Psi_{m}^{k}-\Psi_{m}^{k-1}\right|}{\sum_{m=1}^{N_{m}}\Psi_{m}}\leq \tau \end{array}\right.$$

where *τ* is the tolerance value for the overall topological alteration.

It is known that the SIMP method uses the maximum change in design variables between iterations as the convergence criterion. However, SIMP may face convergence issues when applied to large-scale problems with many elements, as noted in Fu et al. [46]. To overcome this issue, the convergence criterion introduced in Equation (24) relies on a global measure of design variable variation, as opposed to the local criterion typically used in SIMP. The improved effectiveness of this criterion in determining convergence has been demonstrated by Fu et al. [47].

To assess how accurately the level-set function captures the smooth topological boundary, a metric called the topological boundary error is introduced as the second convergence criterion. This error is defined as the proportion of intermediate elements that are not located on the actual boundary relative to the total number of elements. The corresponding convergence criterion for the topological boundary error is defined as

(25) $$\left\{\begin{array}{c} \varepsilon_{M}=\frac{N_{b}}{N_{M}}\leq \epsilon \\ \varepsilon_{m}=\frac{N_{b}}{N_{m}}\leq \epsilon \end{array}\right.$$

where $\varepsilon_{M}$ and $\varepsilon_{m}$ are the topological boundary error for macro- and microdesigns, respectively. $N_{b}\;$ denotes the number of intermediate elements that do not align with the boundary at each scale, and ϵ represents the prescribed tolerance for the topological boundary error. When this error metric falls below the defined threshold, the intermediate elements are effectively confined to the boundary region, suggesting that the level-set function accurately captures the current design geometry.

In this study, both τ and ϵ are defined as 0.001 according to the original setup in Fu and Rolfe [48], as this value offers a balanced trade-off between computational efficiency and solution accuracy. The optimisation comes to convergence when both criteria in Equations (24) and (25) are met.

## 4. Results

This section provides a comprehensive evaluation of the proposed method. In all tested examples, the microstructure size in all normal directions is set to 0.1. Young’s moduli for solid and void regions are 1.0 and $10^{-6}$, respectively, with a Poisson’s ratio of 0.3.

**Figure 2:**
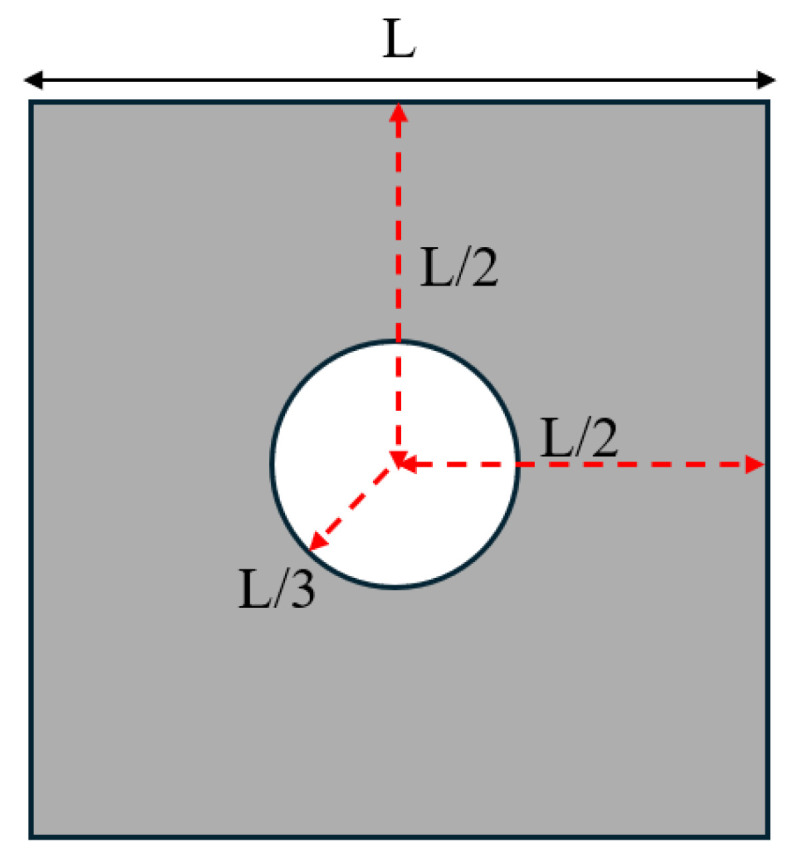
The initial configuration of the microstructures.

It is important to highlight that, throughout the optimisation process, the macroscopic design domain maintains a uniform material distribution to satisfy the target volume fraction. At the same time, the microstructural configurations are initially defined with embedded voids to address the sensitivity uniformity issues typically caused by PBCs. For numerical tests without specific illustrations, the predefined void region is assumed to be a circular area with a radius equal to one-third of the total element length of the microstructure as illustrated in Figure 2, and the number of grid points utilised in the SEMDOT method is set to five.

All numerical experiments were conducted on a computer equipped with an Intel Core i9-14900HK processor, an NVIDIA GeForce RTX 4090 (16 GB, laptop version) graphics card, and 64 GB of RAM, running MATLAB (R2024b).

### 4.1. Messerschmitt–Bölkow–Blohm (MBB) Beam and Michell Beam Design Problems

**Figure 3:**
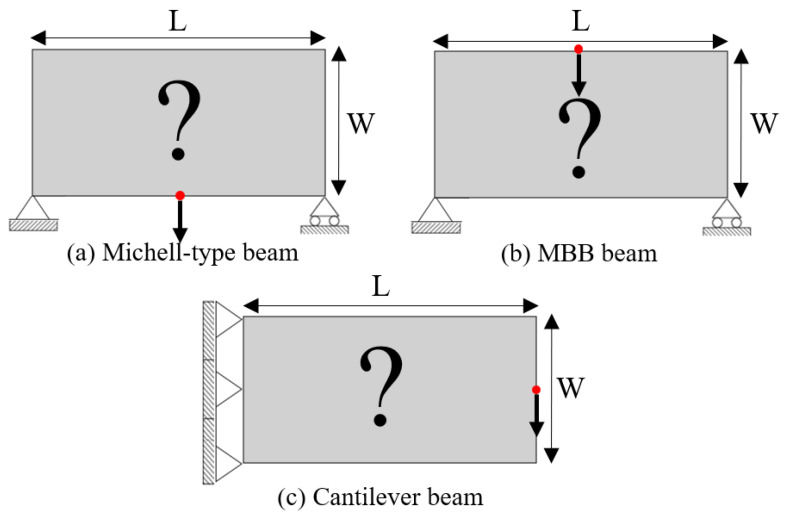
Different types of compliance-based design problems.

In the SEMDOT-based multiscale design, numerical testing begins with a classical Michell-type structure, illustrated in Figure 3a. The loading condition involves a concentrated force at the midpoint of the bottom edge, while the boundary conditions fix the lower right corner and apply a roller support to the lower left corner. The domain dimensions are specified as L=10 and W=4. For the macroscale analysis, a mesh of 100×40 four-node plane stress elements are utilised, and the microscale is discretised using a 50×50 finite mesh. The target volume fractions for the macro- and microscales are 0.4 and 0.6, respectively. The filter radius in the SEMDOT for the macroscale is defined as $r_{min}=2.0h$ and $r_{nmin}=2.0h$, with h indicating the size of a macroelement. For the microscale, the filter radius is defined as $r_{min}=0.04$ and $r_{nmin}=0.04h$.

Table 1 presents a comparative analysis of multiscale designs of the Michell-type beam generated by the SEMDOT method and the SIMP method. Significant differences are observed in the microscopic structural features. Specifically, the designs obtained by the SIMP method in Gao et al. 2019 [33] exhibited numerous intermediate-density elements, whereas the SEMDOT approach produced structures with clearly defined boundaries.

The presence of intermediate elements in the microscopic field in SIMP-based design can be attributed to the applied projection technique in Gao et al. [33], which thresholds the design variables into binary values. This process is governed by an adjustable parameter β that controls the steepness of the projection. Although the convergence criteria are met after 482 iterations, the value of β is still not large enough to fully enforce the design toward a more binary-like distribution, leading to the existence of excessive intermediate elements in the microstructure.

**Figure 4:**
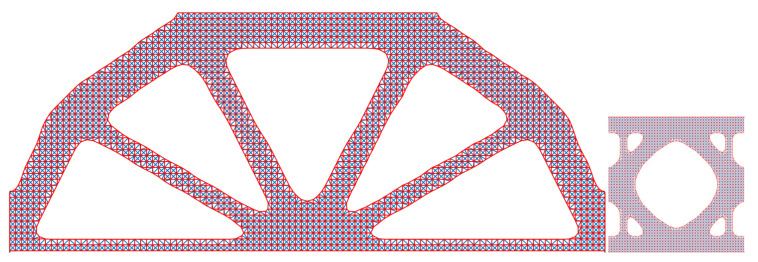
Discretisation of the smooth SEMDOT designs for the Michell structure through a re-meshing approach.

It is noteworthy that the compliance value and homogenised elastic tensor reported for the SEMDOT method are related to the background grayscale design rather than smooth designs [12]. To improve accuracy in evaluating smooth boundary representations, the results of smooth designs presented in the second row of Table 1 are calculated using a re-meshing technique proposed by Zhou et al. [41], which was built on the MATLAB PDE toolbox. This technique generates finite element meshes precisely conforming to smooth boundaries, as illustrated in Figure 4.

The recalculated results for the smooth designs closely match the initial grayscale design results, indicating minimal deviations. Compared to the SIMP method, the SEMDOT method achieves better results with lower compliance values, clearer boundaries, and less than 20% of the total computational time.

The MBB design problem is also studied for further comparison between the two methods. The length and width of the design domain are L=15 and H=3; the boundary and load conditions are shown in Figure 3b. To discretise the macrostructure, 150×30 finite elements are utilised, and the microstructure finite elements remain at 50×50. The target volume fractions of the macro- and microfields are both 40%. In the SEMDOT, the filter radius for the macroscale is set at $r_{min}=2.0h$ and $r_{nmin}=2.0h$, whereas for the microscale, it is established as $r_{min}=0.04h$ and $r_{nmin}=0.04h$.

**Figure 5:**
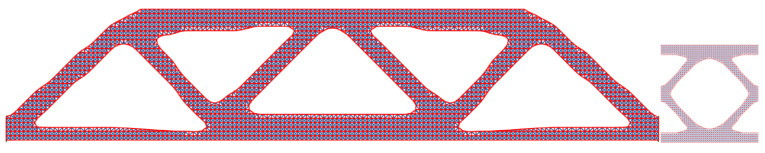
Discretisation of the smooth SEMDOT designs for the MBB beam through a re-meshing approach.

Using the same re-meshing approach, finite element meshes for the smooth multiscale designs are depicted in Figure 5. In Table 2, it can be seen that the results of grayscale and smooth designs based on the SEMDOT method are consistent. Notably, despite similar microstructure designs, SIMP-based macrodesigns differ significantly from those of the SEMDOT, with generally higher compliance values.

It can also be observed that the microscopic design generated by the code used in [33] is closer to a binary design, with fewer intermediate elements compared to the Michell-type beam test case. This indicates that the thresholding process is more efficient with a larger value of β in this case.

Results from both the Michell-type beam and MBB beam affirm that the SEMDOT method consistently outperforms the SIMP method, characterised by smoother boundaries, lower compliance values, and improved computational efficiency. Although the SIMP method performs well in specific tested cases, as validated in Gao et al. [33], the precise control of the projection process, along with the associated computational cost, can pose challenges in more complex design problems.

#### Comparison of State-of-the-Art Multiscale Methods

To further verify the feasibility of applying the SEMDOT to multiscale TO, this section compares the optimisation progress and results of several multiscale algorithms proposed in recent years [33,34,36] when applied to the same cantilever problem. The cantilever beam shown in Figure 3c is tested, where a force of 1.0 is applied at the middle of the right edge, while the left side is fixed. The prescribed domain dimensions are L=10 and W=5. The numerical analysis employs 100×50 four-node plane stress finite elements, while the microscale finite mesh is 50×50. The target volume fractions for the macro- and microscales are 0.4 and 0.5, respectively. For consistency across all methods, the filter radius is set to $r_{min}=2.0h$ at the macroscale and $r_{min}=0.04h$ at the microscale. The initial configuration of microstructure is defined as Figure 2.

**Figure 6:**
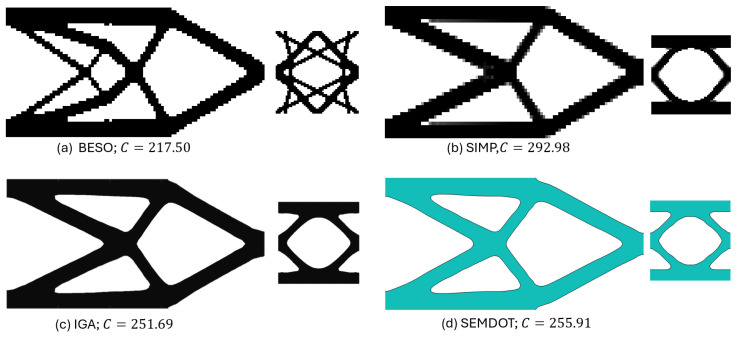
Multiscale design results for the cantilever beam based on (**a**) BESO method, (**b**) SIMP method, (**c**) IGA method, and (**d**) SEMDOT method.

From Figure 6, it can be observed that the resulting macro- and microstructures based on the BESO method differ significantly from those produced by the other methods, while also achieving the lowest compliance value. However, the minimum feature size of the structure violates the prescribed filter radius $r_{min}$, and the presence of jagged boundaries renders the design unsuitable for practical applications. Therefore, additional post-processing, such as applying the strategy in [49], is needed. In contrast, the other three methods generate structures with nearly identical features. The compliance values obtained by the SEMDOT-based and IGA-based methods are comparable, with 255.91 ≳ 251.69, both significantly outperforming the compliance value produced by the SIMP-based method (292.98).

**Figure 7:**
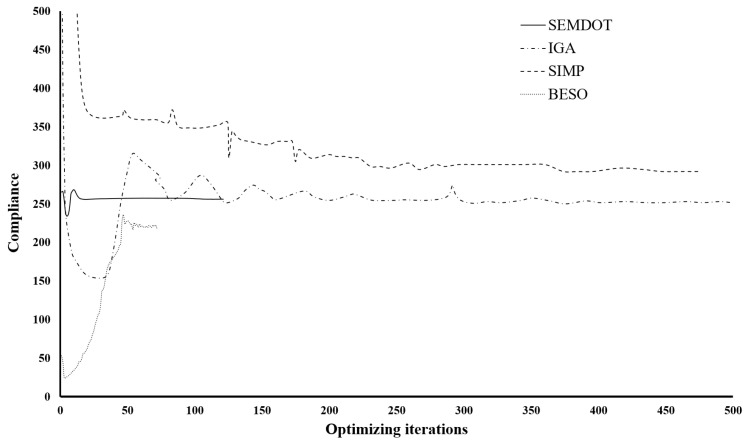
The overall optimisation process under different methods.

Figure 7 presents the optimisation histories of the different methods. The convergence curves for the BESO, IGA, and SIMP methods are taken from [33,34,36]. It can be observed that BESO achieves convergence within seventy-seven iterations, which is substantially fewer than the other three approaches. Among the SEMDOT, IGA, and SIMP methods, the SEMDOT demonstrates the most stable convergence behaviour, reaching convergence after 121 iterations. In contrast, the SIMP and IGA methods exhibit more pronounced fluctuations throughout the first 300 iterations, before converging after more than 450 iterations.

Combining the results from Figure 6 and Figure 7, the SEMDOT demonstrates its effectiveness in generating smooth boundary structures while achieving favourable convergence behaviour and competitive compliance values compared to other state-of-the-art MSTO methods.

### 4.2. Test Different Design Volume Fractions Using the Cantilever Beam Design Problem

This section evaluates the influence of different target volume fractions on overall structural performance. A cantilever beam, as depicted in Figure 3c, is examined again. An external force of F=−1 is applied at the midpoint of the right side. The size of the design domain is specified as L = 10 and W = 4. The macrostructure is discretised into 100 × 40 finite elements, while the microstructure is represented by 50 × 50 finite elements. The filtering radius for the macroscale is set at $r_{min}=1.5h$ and $r_{nmin}=1.0h$, whereas for the microscale, it is established as $r_{min}=0.03h$ and $r_{nmin}=0.02h$. The product of the macrovolume fraction ($V_{M}$) and microvolume fraction ($V_{m}$) is fixed at $V_{M}\times V_{m}=0.2$ to ensure a consistent total material proportion throughout the structure.

Table 3 illustrates a gradual decline in compliance values, suggesting that the single-scale design at the macroscale markedly surpasses its microscale counterpart. These results align with prior research [50,51]. Nevertheless, this does not diminish the relevance of microstructural design, as the present analysis focuses solely on the structure’s overall stiffness. In other contexts, such as energy absorption in composite materials or the creation of porous structures for biomedical applications [52,53], microstructural design can significantly influence performance.

### 4.3. Parametric Study of the SEMDOT Method in Multiscale Design

After validating the applicability of the SEMDOT method in multiscale design, this section analyses the impacts of key parameters on the optimised results. Specifically, Section 4.3.1 examines the effect of microstructural mesh size, while Section 4.3.2 investigates the impact of the number of grid points on the final design.

#### 4.3.1. The Mesh Independency of the SEMDOT Method

This section examines the impact of varying the number of micro-finite elements. The prescribed dimensions for the Michell beam are L = 8 and H = 4. The numerical analysis employs 80 × 40 finite elements for the macrostructure, while the microscale finite elements are increased from 40×40 to 160×160. The target volume fractions for both the macro- and microscales are set to 0.5. The filter radius for the macrostructure is $r_{min}=2.0h$ and $r_{nmin}=1.0h$, whereas for the microstructure, it is rmin=0.05h and $r_{nmin}=0.025h$, where *h* is the size of one macroelement.

In Table 4, It can be concluded that the multiscale design based on the SEMDOT method is reasonably mesh-independent. Although the microdesign with 40 × 40 finite elements includes different hole sizes and positions compared to other mesh sizes, the overall topological structures under the four mesh sizes are consistent, with similar compliance values.

Additionally, unlike single-scale TO, where increasing the mesh size typically leads to higher compliance values, increasing the mesh size at the microscale has minimal impact on compliance values.

#### 4.3.2. The Choice of Number of Grid Points in SEMDOT Method

The grid points are evenly distributed within the unit cell, as shown in Figure 1. Increasing the number of grid points enhances the smoothness of boundary representation. To evaluate the impact of grid numbers, a simple cantilever beam design is used for verification. The beam has dimensions of L=10 and W=5, which is discretised into 100×40 finite elements. The macroscale and microscale design volume fractions are both 0.4, with the microstructure represented by 50 × 50 finite elements. The filter radius for the macroscale is set at $r_{min}=1.5h$ and $r_{nmin}=1.0h,$ whereas for the microscale, the filter radius is $r_{min}=0.03h$ and $r_{nmin}=0.02h$. The number of grid points has progressively increased from 3 to 20.

As shown in Table 5, the number of grid points has a minimal impact on compliance values. Although increasing the number of grid points enables smoother boundary representations, this improvement comes at the cost of significantly higher computational expense. In general, selecting five to ten grid points is recommended, as this range provides a balance between generating smooth structures while maintaining computational efficiency.

#### 4.3.3. The Computational Cost for Different Problems

**Figure 8:**
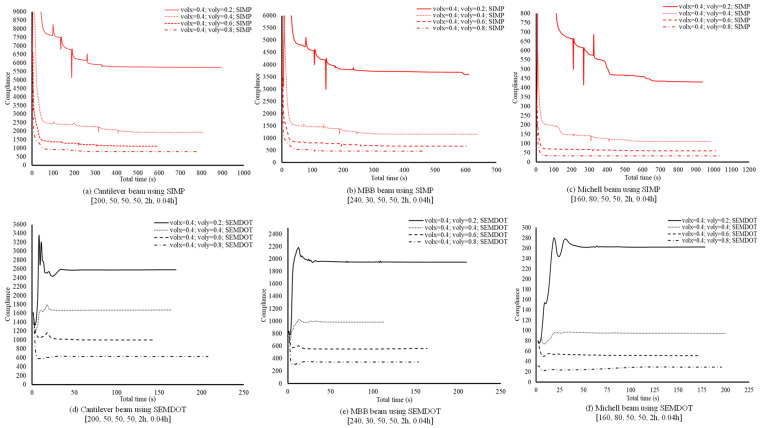
The total computational time of multiscale design using the SEMDOT and SIMP methods. $\left[Nelx,\;Nely,\;nelx,\;nely,\;R_{min},\;r_{min}\right]$ represent the fundamental parameters used in the test cases. $\left(Nelx\times Nely\right)$ defines the size of the macroscale finite element mesh, while $\left(nelx\times nely\right)$ corresponds to the mesh size at the microscale. $R_{min}$ and $r_{min}$ indicate the filter radii applied to the macro- and microdesign fields, respectively.

The computational costs of multiscale design using the SEMDOT method are further investigated and compared with the well-established SIMP-based approach. Three benchmark problems are tested: cantilever, MBB, and Michell beams. As shown in Figure 8, volx and voly represent the target volume fractions at the macro- and microscales, respectively.

The results in Figure 8 demonstrate that the SEMDOT consistently requires less total computational time to achieve stable compliance values, typically around 50 s for all tested cases. In contrast, the SIMP method exhibits larger fluctuations and greater variability across different problems. For instance, at voly=0.2 or 0.4, the SIMP approach requires 300 s to 700 s to stabilise, depending on the case. Even at higher voly values (0.6 or 0.8), convergence times range from 50 s to 200 s. Moreover, the SEMDOT achieves statistically significant compliance improvements across all problem setups compared to SIMP, particularly when lower microstructural volume fractions are used.

### 4.4. Multiscale Design Based on Different Initial Designs of Microstructures

In this section, multiscale design based on different initial designs of microstructures will be discussed, and four different initial designs will be investigated. The radius of all the void circles is defined as 1/8 of the total element length for the square microstructure, as displayed in Figure 8. The validation will be processed on the MBB beam with the same design domain as Section 4.1. For the macrostructure, 150 × 30 finite elements are used, with 50 × 50 for the microstructure. The target volume fractions for the macro- and microscales are 0.6 and 0.5, respectively. The filter radius for the macroscale is $r_{min}=2.5h$ and $r_{nmin}=1.0h$, whereas for the microscale, $r_{min}=0.05h$ and $r_{nmin}=0.02h$.

In Table 6, it is evident that the optimised structural compliances in all cases are nearly equal, despite the differences in material microstructure topologies. This phenomenon primarily arises from the non-uniqueness inherent to the optimisation of material microstructures, as documented in prior studies [30,33]. As presented in the third column of Table 6, the homogenised elastic tensors in all cases exhibit close similarity, and the optimised macroscopic topologies are also similar. Consequently, we can assert that the initial microstructure design has a negligible impact on the macroscopic topological design and mechanical performance although it can significantly impact microstructure design.

### 4.5. Extension to Three-Dimensional Design Problems

**Figure 9:**
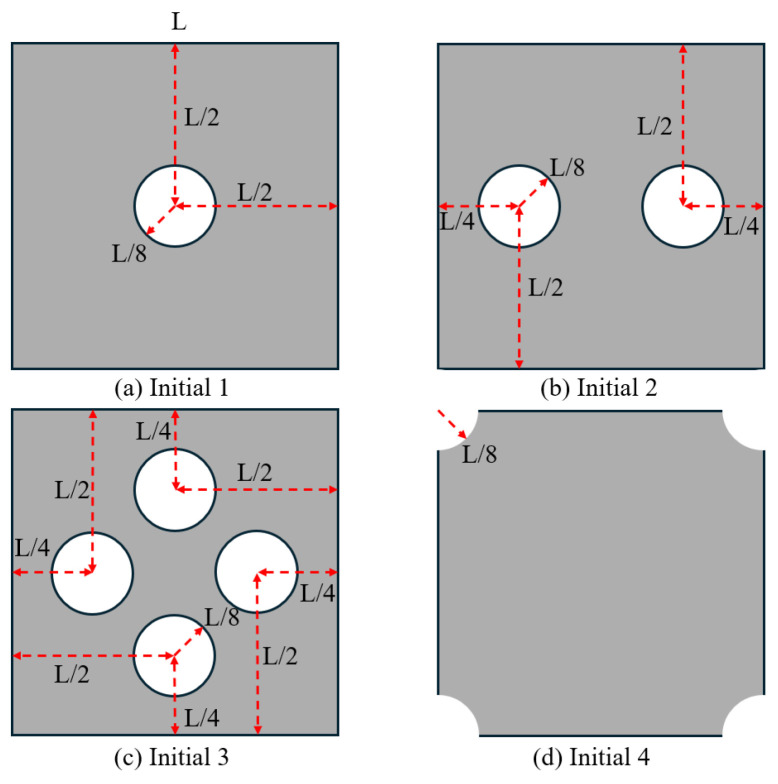
Different initial designs for microstructures.

In addition to the 2D test cases, the multiscale design using the SEMDOT method can be extended to 3D design problems. The first test case shown in Figure 9 corresponds to the 3D-supported structure test case. The macrodesign domain has dimensions L = 2, H = 1.6, and W = 2, and is discretised into 20 × 16 × 20 finite elements. For the microstructure, a periodic microunit cell with a square geometry is considered, discretised into 10 × 10 × 10 finite elements along three directions. The load with a value of 1.0 is applied on the middle point of the bottom surface, while the material properties are assumed isotropic, with a Young’s modulus of 1.0 and Poisson’s ratio of 0.3. To help with convergence in the three-dimensional case, the maximum iteration is set to 150. The target volume fractions are 20% for the 3D-supported structure and 30% for the cantilever test case. The filtering process employs both an elemental density filter and a nodal filter, with $r_{min}=2.0h$ and $r_{nmin}=2.0h$ for the macroscale, respectively. For the microscale, $r_{min}=0.04h$ and $r_{nmin}=0.04h$.

**Figure 10:**
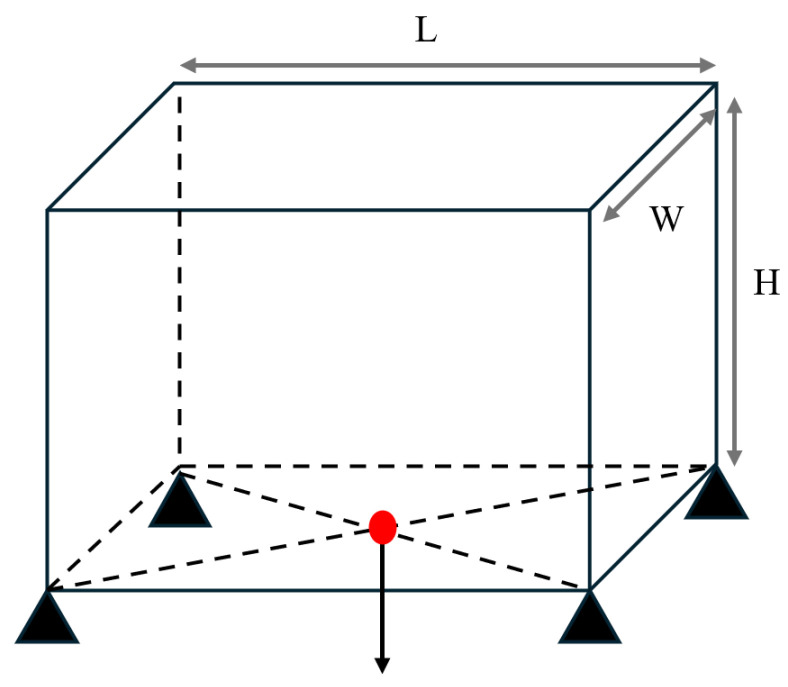
The load and boundary conditions of a 3D-supported structure.

Additionally, a three-dimensional MBB beam is optimised. Its design domain dimensions are L = 12, H = 2, and W = 1, as illustrated in Figure 10. The macroscale is discretised into 120×20×10 eight-node cubic finite elements, while the microscale is discretised into 10 × 10 × 10 cubic finite elements. The design volume fractions are set to 0.3 and 0.5 for the macro- and microscales, respectively. The loading condition is applied at the midpoint of the top surface, while red arrows at four bottom vertices indicate fixed degrees of freedom.

**Figure 11:**
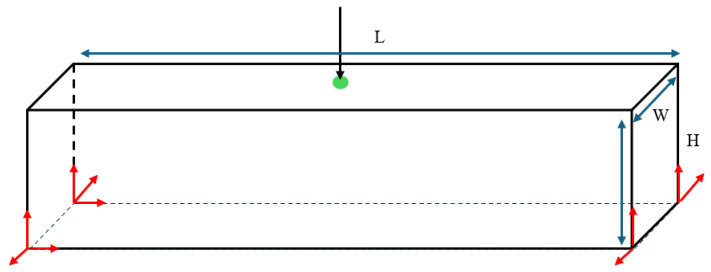
The load and boundary conditions of a three-dimensional MBB beam.

The initial microstructure designs for both test cases are shown in Figure 11. The first configuration consists of eight octant-sphere void regions located at each vertex of the microunit cell. The second configuration features a single spherical void region positioned at the inside centre of the unit cell. The radius is one-third of the total microelement size L. The optimised geometries obtained for these cases are presented in Table 7 and Table 8.

**Table 7:** The multiscale design results for a 3D-supported structure based on two different initial microconfigurations.

Design results based on both scales based on initial microstructure in Figure 12a	
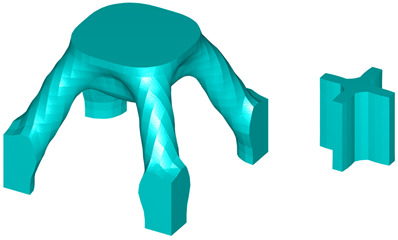	
Homogenised elastic modulus	Total Compliance
$\left[\begin{array}{cccccc} 0.1881 & 0.0625 & 0.0204 & 0 & 0 & 0\\ 0.0625 & 0.3375 & 0.0625 & 0 & 0 & 0\\ 0.0204 & 0.0625 & 0.1881 & 0 & 0 & 0\\ 0 & 0 & 0 & 0.0663 & 0 & 0\\ 0 & 0 & 0 & 0 & 0.0663 & 0\\ 0 & 0 & 0 & 0 & 0 & 0.0112 \end{array}\right]$	182.8589
Design results based on both scales based on initial microstructure in Figure 12b	
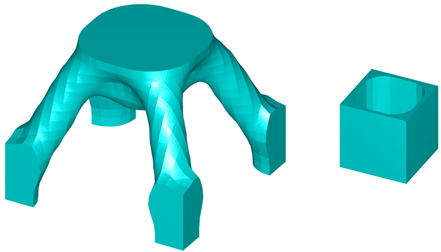	
Homogenised elastic modulus	Total Compliance
$\left[\begin{array}{cccccc} 0.1881 & 0.0625 & 0.0204 & 0 & 0 & 0\\ 0.0625 & 0.3375 & 0.0625 & 0 & 0 & 0\\ 0.0204 & 0.0625 & 0.1881 & 0 & 0 & 0\\ 0 & 0 & 0 & 0.0663 & 0 & 0\\ 0 & 0 & 0 & 0 & 0.0663 & 0\\ 0 & 0 & 0 & 0 & 0 & 0.0112 \end{array}\right]$	187.282

**Table 8:** The multiscale design results for a 3D MBB beam based on two different initial microconfigurations.

Design results based on both scales based on initial microstructure in Figure 12a	
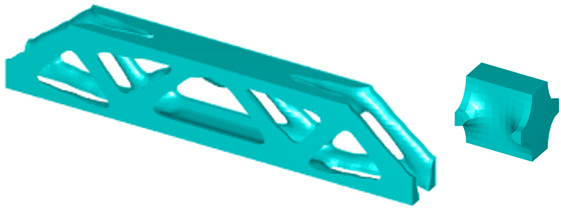	
Homogenised elastic modulus	Compliance
$\left[\begin{array}{cccccc} 0.5208 & 0.1606 & 0.0388 & 0 & 0 & 0\\ 0.1606 & 0.5179 & 0.0377 & 0 & 0 & 0\\ 0.0388 & 0.0377 & 0.1311 & 0 & 0 & 0\\ 0 & 0 & 0 & 0.1773 & 0 & 0\\ 0 & 0 & 0 & 0 & 0.0307 & 0\\ 0 & 0 & 0 & 0 & 0 & 0.0349 \end{array}\right]$	316.0817
Design results based on both scales based on initial microstructure in Figure 12b	
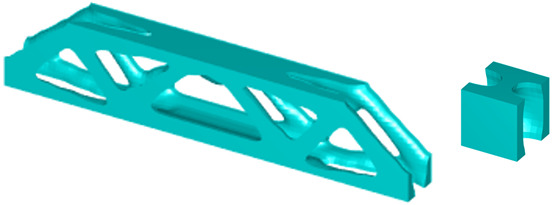	
Homogenised elastic modulus	Compliance
$\left[\begin{array}{cccccc} 0.5206 & 0.1558 & 0.0422 & 0 & 0 & 0\\ 0.1558 & 0.5010 & 0.0402 & 0 & 0 & 0\\ 0.0422 & 0.0402 & 0.1424 & 0 & 0 & 0\\ 0 & 0 & 0 & 0.1724 & 0 & 0\\ 0 & 0 & 0 & 0 & 0.0364 & 0\\ 0 & 0 & 0 & 0 & 0 & 0.0358 \end{array}\right]$	316.1200

**Figure 12:**
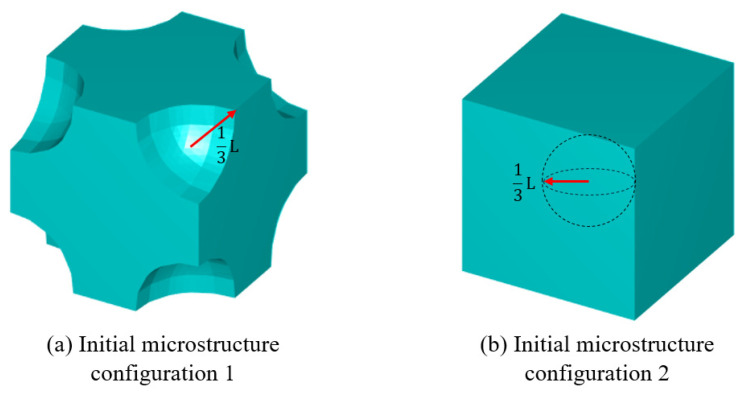
The initial microconfigurations for three-dimensional multiscale design problems.

As indicated by the results in Table 7 and Table 8, the SEMDOT method proves effectiveness in addressing 3D design problems. Both micro- and macrodesigns exhibit smooth and well-defined boundary representations, which are beneficial for manufacturing and computer-aided design. Moreover, similar to the 2D test cases with different initial microstructures, although microtopologies can be significantly impacted, the optimised macrotopologies and structural compliance values remain nearly identical across all cases for both the 3D-supported structure and MBB design problems.

## 5. Conclusions

This study successfully extends the SEMDOT method to MSTO. Through extensive 2D and 3D test cases, the method demonstrates its effectiveness in generating smooth and well-defined structural designs while mitigating common issues such as blurred regions or staircasing boundaries, which are crucial for computer-aided design and manufacturing. Key findings from the study highlight the following:

- The remeshing results confirm that the structural properties of grayscale and smooth SEMDOT designs are highly consistent, indicating minimal deviation introduced by the level-set representation.
- The multiscale cantilever beam design achieved by the SEMDOT is comparable to the results obtained by other state-of-the-art approaches, while requiring fewer iterations to meet convergence criteria than the SIMP and IGA methods.
- The Michell beam problem demonstrates the mesh independence of the SEMDOT in multiscale settings. Increasing the number of grid points per unit cell has little impact on the final design but significantly increases computational cost.
- Across the MBB, cantilever, and Michell beam benchmarks, the SEMDOT consistently demonstrates statistically significant improvements in compliance and reductions in computational cost compared to SIMP-based multiscale designs.
- The SEMDOT framework is successfully extended to both 2D and 3D problems, maintaining smooth boundaries and stable compliance performance across varying initial microstructure topologies.

Despite these advantages, further research is needed to explore the SEMDOT’s adaptability in complex engineering applications, such as compliant mechanisms, heat conduction, natural frequency, and buckling problems. In contrast to stiffness-based objectives, buckling is governed by critical loads and is highly sensitive to design perturbations, as small changes may lead to sudden structural failure. As such, the applicability of the discrete sensitivity scheme in the SEMDOT for buckling-driven optimisation problems warrants further investigation. Additionally, multi-objective multiscale design integrating these challenges presents a promising direction. Overall, this study extends the SEMDOT method to multiscale design, advancing the development of efficient and manufacturable topology-optimised structures.

## Figures and Tables

**Table 1:** A comparison of multiscale designs of a Michell-type beam between the SEMDOT method and the SIMP method.

Method	Macrodesign	Microdesign	Results
SEMDOT grayscale design	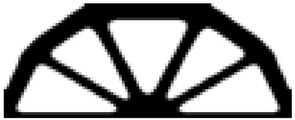	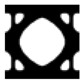	$D^{H}:\left[\begin{array}{ccc} 0.4028 & 0.1179 & 0\\ 0.1179 & 0.3379 & 0\\ 0 & 0 & 0.1212 \end{array}\right]$ Itr: 131 T: 102.574s C: 60.556
SEMDOT smooth design	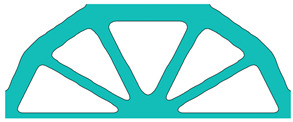	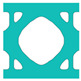	$D^{H}:\left[\begin{array}{ccc} 0.4013 & 0.1186 & 0\\ 0.1186 & 0.3368 & 0\\ 0 & 0 & 0.1204 \end{array}\right]$ C: 60.933
SIMP	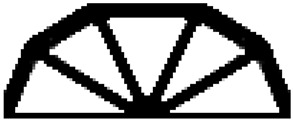	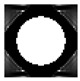	$D^{H}:\left[\begin{array}{ccc} 0.3736 & 0.0998 & 0\\ 0.0998 & 0.3443 & 0\\ 0 & 0 & 0.0824 \end{array}\right]$ Itr: 482 T: 544.998s C: 73.275

$D^{H}$ is the homogenised elastic tensor; Itr is the total iteration to convergence; T is the computational time; C is the compliance value.

**Table 3:** The design for cantilever beams under different groups of design volume fractions.

Design Volume Fraction	Macrodesign	Microdesign	Compliance
$V_{M}=1.0$ $V_{m}=0.2$	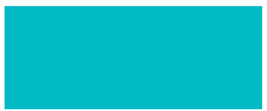	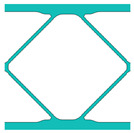	642.3547
$V_{M}=0.8$ $V_{m}=0.25$	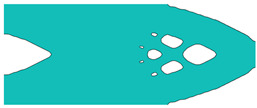	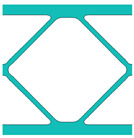	544.0138
$V_{M}=0.6$ $V_{m}=0.333$	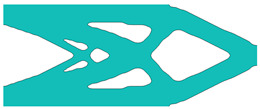	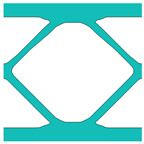	490.7782
$V_{M}=0.4$ $V_{m}=0.5$	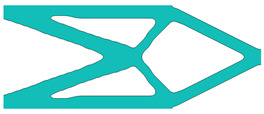	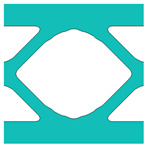	416.2822
$V_{M}=0.2$ $V_{m}=1.0$	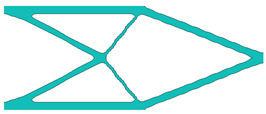	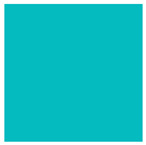	235.7313

**Table 4:** The multiscale design of Michell-type beam with different number of micro-finite elements.

Mesh Size and Filter Radius of Microunit	Macrodesign	Microdesign	Homogenised Elastic Tensor and Total Compliance
40×40	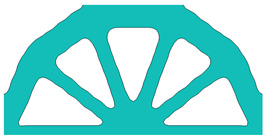	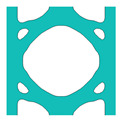	$\left[\begin{array}{ccc} 0.2452 & 0.0908 & 0\\ 0.0908 & 0.3138 & 0\\ 0 & 0 & 0.0858 \end{array}\right]$ Compliance: 55.4930
80×80	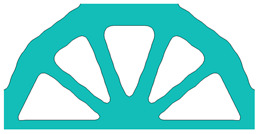	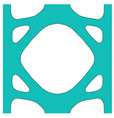	$\left[\begin{array}{ccc} 0.2543 & 0.0963 & 0\\ 0.0963 & 0.2955 & 0\\ 0 & 0 & 0.0961 \end{array}\right]$ Compliance: 54.5808
120 × 120	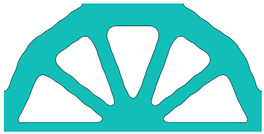	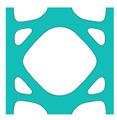	$\left[\begin{array}{ccc} 0.2558 & 0.0962 & 0\\ 0.0962 & 0.2929 & 0\\ 0 & 0 & 0.0955 \end{array}\right]$ Compliance: 54.7190
160 × 160	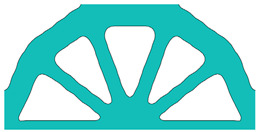	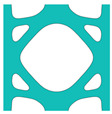	$\left[\begin{array}{ccc} 0.2546 & 0.0955 & 0\\ 0.0955 & 0.2943 & 0\\ 0 & 0 & 0.0942 \end{array}\right]$ Compliance: 54.8811

**Table 5:** The design results and computational cost of cantilever beam problem.

Grid Number	Macroscopic Result	Microscopic Result	Compliance	Homogenised Elastic Tensor	Computational Time (s)
3	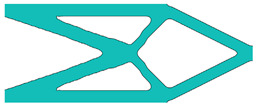	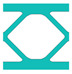	554.585	$\left[\begin{array}{ccc} 0.3143 & 0.0565 & 0\\ 0.0565 & 0.0729 & 0\\ 0 & 0 & 0.0586 \end{array}\right]$	60.544s
5	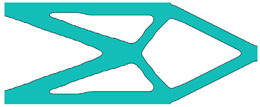	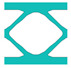	553.465	$\left[\begin{array}{ccc} 0.3192 & 0.0536 & 0\\ 0.0536 & 0.0635 & 0\\ 0 & 0 & 0.0589 \end{array}\right]$	79.987s
10	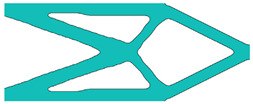	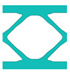	555.118	$\left[\begin{array}{ccc} 0.3198 & 0.0520 & 0\\ 0.0520 & 0.0633 & 0\\ 0 & 0 & 0.0577 \end{array}\right]$	134.016s
20	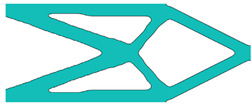	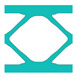	555.736	$\left[\begin{array}{ccc} 0.3200 & 0.0519 & 0\\ 0.0519 & 0.0631 & 0\\ 0 & 0 & 0.0574 \end{array}\right]$	338.204s

**Table 6:** The multiscale design results of MBB beam based on different initial design of microstructures.

Initial Design of Microstructure	Design Results for Both Scales	Homogenised Elastic Tensor and Compliance
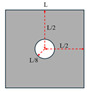	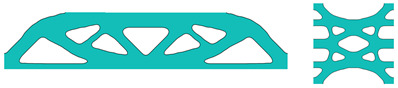	$\left[\begin{array}{ccc} 0.3909 & 0.0749 & 0\\ 0.0749 & 0.1567 & 0\\ 0 & 0 & 0.0810 \end{array}\right]$ Compliance: 182.7206
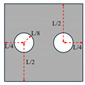	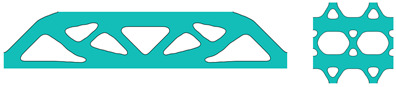	$\left[\begin{array}{ccc} 0.3761 & 0.0679 & 0\\ 0.0679 & 0.2072 & 0\\ 0 & 0 & 0.0718 \end{array}\right]$ Compliance: 183.2987
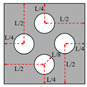	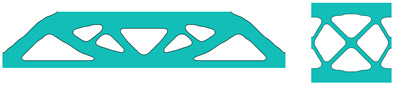	$\left[\begin{array}{ccc} 0.3856 & 0.0791 & 0\\ 0.0791 & 0.1581 & 0\\ 0 & 0 & 0.0854 \end{array}\right]$ Compliance: 182.7502
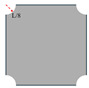	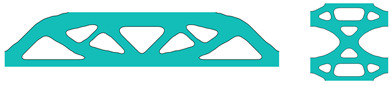	$\left[\begin{array}{ccc} 0.3892 & 0.0751 & 0\\ 0.0751 & 0.1557 & 0\\ 0 & 0 & 0.0823 \end{array}\right]$ Compliance: 182.5309

**Table 2:** A comparison of multiscale designs of the MBB beam between the SEMDOT method and the SIMP method.

Method	Macrodesign	Microdesign	Results
SEMDOT grayscale design	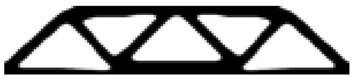	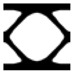	$D^{H}:\left[\begin{array}{ccc} 0.2892 & 0.0628 & 0\\ 0.0628 & 0.0906 & 0\\ 0 & 0 & 0.0621 \end{array}\right]$ Itr:84 T: 98.375s C: 373.372
SEMDOT smooth design	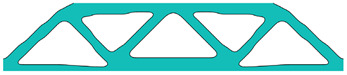	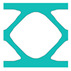	$D^{H}:\left[\begin{array}{ccc} 0.2899 & 0.0628 & 0\\ 0.0628 & 0.0888 & 0\\ 0 & 0 & 0.0614 \end{array}\right]$ C: 372.649
SIMP	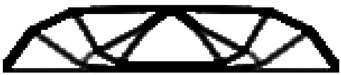	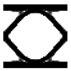	$D^{H}:\left[\begin{array}{ccc} 0.2797 & 0.0537 & 0\\ 0.0537 & 0.0731 & 0\\ 0 & 0 & 0.0513 \end{array}\right]$ Itr: 385 T: 472.687s C: 431.826

$D^{H}$ is the homogenised elastic tensor; Itr is the total iteration to convergence; T is the computational time; C is the compliance value.

## Data Availability

The original contributions presented in this study are included in the article. Further inquiries can be directed to the corresponding author.

## Abbreviations

The following abbreviations are used in this manuscript:

TO	Topology optimisation
MSTO	Multiscale topology optimisation
SEMDOT	Smooth-edged material distribution for optimisation topology
SIMP	Solid isotropic material penalisation
BESO	Bi-directional evolutionary structural optimisation
LSM	Level set method
IGA	Isogeometrical
M-VCUT	Multiple variable cutting
MMC	Moving morphable component
MMV	Moving morphable void
FEA	Finite element analysis
MBB	Messerschmitt–Bölkow–Blohm
RVE	Representative volume element
PBCs	Periodical boundary conditions

## References

[1] Bendsoe M.P., Sigmund O. (2013). Topology Optimization: Theory, Methods, and Applications.

[2] Jihong Z., Han Z., Chuang W., Lu Z., Shangqin Y., Zhang W. (2021). A review of topology optimization for additive manufacturing: Status and challenges. Chin. J. Aeronaut..

[3] Bendsøe M.P. (1989). Optimal shape design as a material distribution problem. Struct. Optim..

[4] Zhou M., Rozvany G.I. (1991). The COC algorithm, Part II: Topological, geometrical and generalized shape optimization. Comput. Methods Appl. Mech. Eng..

[5] Querin O.M., Steven G.P., Xie Y.M. (1998). Evolutionary structural optimisation (ESO) using a bidirectional algorithm. Eng. Comput..

[6] Yang X., Xie Y., Steven G., Querin O. (1999). Bidirectional evolutionary method for stiffness optimization. AIAA J..

[7] Wang M.Y., Wang X., Guo D. (2003). A level set method for structural topology optimization. Comput. Methods Appl. Mech. Eng..

[8] Guo X., Zhang W., Zhong W. (2014). Doing topology optimization explicitly and geometrically—A new moving morphable components based framework. J. Appl. Mech..

[9] Zhang W., Chen J., Zhu X., Zhou J., Xue D., Lei X., Guo X. (2017). Explicit three dimensional topology optimization via Moving Morphable Void (MMV) approach. Comput. Methods Appl. Mech. Eng..

[10] Zhang X., Takezawa A., Kang Z. (2019). Robust topology optimization of vibrating structures considering random diffuse regions via a phase-field method. Comput. Methods Appl. Mech. Eng..

[11] Fujioka M., Shimoda M., Al Ali M. (2022). Concurrent shape optimization of a multiscale structure for controlling macrostructural stiffness. Struct. Multidiscip. Optim..

[12] Fu Y.-F., Long K., Rolfe B. (2023). On non-penalization SEMDOT using discrete variable sensitivities. J. Optim. Theory Appl..

[13] Fu Y.-F., Ghabraie K., Rolfe B., Wang Y., Chiu L.N. (2021). Smooth design of 3D self-supporting topologies using additive manufacturing filter and semdot. Appl. Sci..

[14] Fu Y.-F., Rolfe B. (2023). Non-penalization topology optimization for maximizing natural frequency using SEMDOT. Proceedings of the IASS Annual Symposia.

[15] Zhang K., Li B., Du F., Liu H., Hong J. (2021). Topology optimization of natural convection heat transfer using SEMDOT algorithm based on the reduced-order model. Int. Commun. Heat. Mass. Transf..

[16] Ribeiro T., Fu Y.-F., Bernardo L., Rolfe B. (2023). Topology Optimisation of Structural Steel with Non-Penalisation SEMDOT: Optimisation, Physical Nonlinear Analysis, and Benchmarking. Appl. Sci..

[17] Kumar A., Nune K., Murr L., Misra R. (2016). Biocompatibility and mechanical behaviour of three-dimensional scaffolds for biomedical devices: Process–structure–property paradigm. Int. Mater. Rev..

[18] Wu J., Sigmund O., Groen J.P. (2021). Topology optimization of multi-scale structures: A review. Struct. Multidiscip. Optim..

[19] Bendsøe M.P., Kikuchi N. (1988). Generating optimal topologies in structural design using a homogenization method. Comput. Methods Appl. Mech. Eng..

[20] Allaire G., Brizzi R. (2005). A multiscale finite element method for numerical homogenization. Multiscale Model. Simul..

[21] Wu Z., Xia L., Wang S., Shi T. (2019). Topology optimization of hierarchical lattice structures with substructuring. Comput. Methods Appl. Mech. Eng..

[22] Wang C., Zhu J.H., Zhang W.H., Li S.Y., Kong J. (2018). Concurrent topology optimization design of structures and non-uniform parameterized lattice microstructures. Struct. Multidiscip. Optim..

[23] Imediegwu C., Murphy R., Hewson R., Santer M. (2019). Multiscale structural optimization towards three-dimensional printable structures. Struct. Multidiscip. Optim..

[24] Feng J., Wang L., Zhai X., Chen K., Wu W., Liu L., Fu X.-M. (2025). Constructing boundary-identical microstructures via guided diffusion for fast multiscale topology optimization. Comput. Methods Appl. Mech. Eng..

[25] Zhai X., Wang W., Chen F., Wu J. (2024). Topology optimization of differentiable microstructures. Comput. Methods Appl. Mech. Eng..

[26] Shao M., Xia Q. (2024). Concurrent Topology Optimization of Two–Scale Structures Based on M–VCUT Level Set Model. Proceedings of the International Conference on Computational & Experimental Engineering and Sciences.

[27] Liu H., Zong H., Shi T., Xia Q. (2020). M-VCUT level set method for optimizing cellular structures. Comput. Methods Appl. Mech. Eng..

[28] Guedes J., Kikuchi N. (1990). Preprocessing and postprocessing for materials based on the homogenization method with adaptive finite element methods. Comput. Methods Appl. Mech. Eng..

[29] Hashin Z. (1983). Analysis of composite materials—A survey. J. Appl. Mech..

[30] Xia L., Breitkopf P. (2015). Design of materials using topology optimization and energy-based homogenization approach in Matlab. Struct. Multidiscip. Optim..

[31] Zhang G., Khandelwal K. (2019). Computational design of finite strain auxetic metamaterials via topology optimization and nonlinear homogenization. Comput. Methods Appl. Mech. Eng..

[32] Andreassen E., Andreasen C.S. (2014). How to determine composite material properties using numerical homogenization. Comput. Mater. Sci..

[33] Gao J., Luo Z., Xia L., Gao L. (2019). Concurrent topology optimization of multiscale composite structures in Matlab. Struct. Multidiscip. Optim..

[34] Kazakis G., Lagaros N.D. (2023). Multi-Scale Concurrent Topology Optimization Based on BESO, Implemented in MATLAB. Appl. Sci..

[35] Sigmund O. (2007). Morphology-based black and white filters for topology optimization. Struct. Multidiscip. Optim..

[36] Aminzadeh M., Tavakkoli S.M. (2024). Multiscale topology optimization of structures by using isogeometrical level set approach. Finite Elem. Anal. Des..

[37] Liang Y., Cheng G. (2019). Topology optimization via sequential integer programming and canonical relaxation algorithm. Comput. Methods Appl. Mech. Eng..

[38] Liang Y., Cheng G. (2020). Further elaborations on topology optimization via sequential integer programming and Canonical relaxation algorithm and 128-line MATLAB code. Struct. Multidiscip. Optim..

[39] Liang Y., Sun K., Cheng G. (2020). Discrete variable topology optimization for compliant mechanism design via Sequential Approximate Integer Programming with Trust Region (SAIP-TR). Struct. Multidiscip. Optim..

[40] Liang Y., Yan X., Cheng G. (2022). Explicit control of 2D and 3D structural complexity by discrete variable topology optimization method. Comput. Methods Appl. Mech. Eng..

[41] Zhou J., Wang Y., Chiu L.N., Ghabraie K. (2025). On the suitability of simplified sensitivity estimation for partial elements in topology optimization. Eng. Optim..

[42] Huang X., Xie Y. (2007). Convergent and mesh-independent solutions for the bi-directional evolutionary structural optimization method. Finite Elem. Anal. Des..

[43] Sigmund O., Aage N., Andreassen E. (2016). On the (non-) optimality of Michell structures. Struct. Multidiscip. Optim..

[44] Sigmund O. (1994). Materials with prescribed constitutive parameters: An inverse homogenization problem. Int. J. Solids Struct..

[45] Svanberg K. (2007). MMA and GCMMA-two methods for nonlinear optimization. Mathematics.

[46] Fu Y.-F., Rolfe B., Wang Y., Huang X., Ghabraie K. (2020). SEMDOT: Smooth-edged material distribution for optimizing topology algorithm. Adv. Eng. Softw..

[47] Fu Y.-F., Rolfe B., Chiu L.N., Wang Y., Huang X., Ghabraie K. (2020). Parametric studies and manufacturability experiments on smooth self-supporting topologies. Virtual Phys. Prototyp..

[48] Fu Y.-F., Rolfe B. (2025). Smooth Topological Design of Continuum Structures.

[49] Li Z., Lee T.-U., Yao Y., Xie Y.M. (2022). Smoothing topology optimization results using pre-built lookup tables. Adv. Eng. Softw..

[50] Vicente W., Zuo Z., Pavanello R., Calixto T., Picelli R., Xie Y. (2016). Concurrent topology optimization for minimizing frequency responses of two-level hierarchical structures. Comput. Methods Appl. Mech. Eng..

[51] Sivapuram R., Dunning P.D., Kim H.A. (2016). Simultaneous material and structural optimization by multiscale topology optimization. Struct. Multidiscip. Optim..

[52] Zhang H., Wang Y., Kang Z. (2019). Topology optimization for concurrent design of layer-wise graded lattice materials and structures. Int. J. Eng. Sci..

[53] Wu J., Aage N., Westermann R., Sigmund O. (2017). Infill optimization for additive manufacturing—Approaching bone-like porous structures. IEEE Trans. Vis. Comput. Graph..

